# Use of sensing technologies to assess sleep quality and physical activity levels in nursing home residents with dementia taking nightly psychotropic drugs for sleep disturbance: a cross-sectional study

**DOI:** 10.3389/fragi.2026.1768935

**Published:** 2026-02-25

**Authors:** Lydia D. Boyle, Brice Marty, Kristoffer Haugarvoll, Ole Martin Steihaug, Bettina S. Husebo

**Affiliations:** 1 Department of Global Public Health and Primary Care, Centre for Elderly and Nursing Home Medicine, University of Bergen, Bergen, Norway; 2 Department of Neurology, Neuro-SysMed, Haukeland University Hospital, Bergen, Norway; 3 Department of Clinical Medicine, Neuro-SysMed, University of Bergen, Bergen, Norway; 4 Department of Internal Medicine, Haraldsplass Deaconess Hospital, Bergen, Norway; 5 Department of Clinical Science, University of Bergen, Bergen, Norway

**Keywords:** dementia, nursing home, physical activity, psychotropic drugs, sensing technology, sleep disturbance

## Abstract

**Background:**

Sleep disturbances are common in people with dementia and nightly prescribed psychotropic drugs, such as sedatives or antidepressants, can increase risks such as injury, inactivity, and behavioral symptoms. Treatment decisions currently rely on periodic, proxy-rated questionnaires that may miss important daily fluctuations in sleep and activity. We explore whether sensing technologies provide insights into distinct differences in sleep characteristics and activity levels in nursing home residents with dementia who are prescribed nightly psychotropic drugs for sleep disturbances.

**Methods:**

Forty-seven participants were recruited from four nursing homes in Bergen, Norway, and stratified according to prescribed nightly psychotropic drug use for sleep disturbance: 1) none, 2) medications with short half-lives, 3) medications with long half-lives. Garmin Vivoactive5 and Venu3, Vital Things Somnofy sleep monitor, and traditional questionnaires (Physical Self Maintenance Scale and Neuropsychiatric Inventory-Nursing Home version) were used for data collection. Digital metrics included Euclidean Norm Minus One (ENMO; day/night/24-h), Sleep Regulatory Index (SRI), Sleep Efficiency (SE), Total Sleep Time (TST), Sleep Fragmentation Index (SFI), and no presence (time out of bed).

**Results:**

Thirty participants (73–100 years old) were included for analysis. Groups taking psychotropic medications were awake for longer periods (WASO: chi^2^ = 8.7, p = 0.01) and had poorer sleep regularity (SRI: chi^2^ = 20.6, p = 0.0001). Participants taking psychotropic drugs had less physical activity (day/night/24-h ENMO), with greatest differences between those on medications with a long half-life (day: chi^2^ = 9.48, p = 0.009; night: chi^2^ = 12.83, p = 0.002; 24-h: chi^2^ = 8.23, p = 0.02) and those not on nightly psychotropic medications.

**Conclusion:**

The digital biomarkers collected using the selected sensing technologies offered nuanced information regarding sleep behaviors and physical activity levels, providing detailed distinction between the groups. Sensing technologies may be a promising companion to the currently used proxy-rated assessment tools for sleep disturbance and physical activity levels for people with dementia residing in nursing homes.

## 1 Introduction

Sleep disturbance and reduced physical activity levels are highly prevalent among individuals with dementia and contribute to poorer health outcomes, accelerated cognitive decline, and increased caregiver strain (Shi et al., 2018). Pharmacological management, particularly psychotropic drug use, remains common for treating sleep problems despite concerns about overuse, limited evidence of effectiveness, and the risk of adverse effects (Watt et al., 2024). Currently, sleep disturbance and activity changes are assessed through periodic proxy-rated questionnaires, which often fail to capture day-to-day variability or the subtle progression of symptoms (Lopez-Garcia et al., 2023; VandeBunte et al., 2022). These limitations reduce clinicians’ ability to identify early deterioration or evaluate the impact of pharmacological and nonpharmacological interventions.

Recent advancements in sensor technologies offer new opportunities to improve monitoring of sleep and physical activity level changes in nursing home settings. Wearable accelerometers, environmental sensors, and radar-based technology provide continuous, objective data that can quantify activity patterns, sleep disruption, and circadian rhythm disturbances with minimal burden on residents (Au-Yeung et al., 2022; Schutz et al., 2021). These technologies have the potential to bridge the gap between the gold standard precision of polysomnography (PSG) and the practicality required in a nursing home setting. Sensing and radar technologies offer advantages over currently used proxy-rated questionnaires by providing real-time, continuous, and unobtrusive monitoring of activity and sleep whereas traditional outcome measures, such as the Neuropsychiatric Inventory (NPI), are only performed periodically and retrospectively, creating increased risk for biases and low validity.

Sleep quality declines with age, with older adults experiencing more awakenings, reduced sleep duration, and diminished deep sleep (Li et al., 2018). Among people with dementia living in nursing homes, sleep quality is especially poor, with up to 70% experiencing sleep disturbances during the disease trajectory (Webster et al., 2020) and is closely associated with behavioral and psychological symptoms of dementia (BPSD), including agitation, depression, psychosis, and pain (Shin et al., 2014). Common sleep problems in this population include insomnia, sundowning, restless legs, difficulty initiating and maintaining sleep, and fragmented nighttime sleep (Wennberg et al., 2017). The autonomic nervous system (ANS), composed of the sympathetic and parasympathetic branches, plays a central role in sleep regulation and is affected by both advanced age and neurodegeneration. In older adults, particularly those with neurodegenerative disease, the normal parasympathetic dampening of sympathetic activity during deep sleep is reduced, contributing to ANS dysregulation and greater sleep disturbances (Liu et al., 2024). Individuals with dementia show even lower parasympathetic activity compared to healthy older adults, which may exacerbate common disease-related symptoms (Liu et al., 2024). Notably, sleep durations of 6–9 h per night have been associated with increased parasympathetic activity in older people with neurodegenerative conditions (Liu et al., 2024). For assessment of sleep disorders, a variety of proxy-rated questionnaires are commonly used within nursing home settings. PSG remains the gold standard for diagnosis of sleep disorders (Boulos et al., 2019). However, its use in nursing homes is limited because the procedure is intrusive, resource-intensive, and often poorly tolerated by older people with dementia (Bombois et al., 2010).

Because individuals with dementia often live with multiple comorbidities and experience complex sleep disturbances, nonpharmaceutical interventions are recommended as first-line treatments (De Bondt et al., 2025). Psychotropic medications can increase risks of injury, worsen BPSD, and lead to side effects that further disturb sleep (Brimelow et al., 2019; Wetzels et al., 2011). A recent longitudinal study showed that extended daytime use of psychotropic drugs increased sedentary behavior and decreased overall physical activity, contributing to poorer sleep (Lim et al., 2023). Evidence supporting pharmacological treatments for sleep disorders in dementia remains limited (McCleery and Sharpley, 2020), yet antipsychotics, benzodiazepines, melatonin, sedating antidepressants, antihistamines, and Z-drugs are commonly prescribed (Gulla et al., 2016). These medications have varied half-lives and may cause next-day grogginess, slowed cognition, and increased fall risk, especially in older adults with altered drug metabolism and polypharmacy (Gulla et al., 2016).

Inactivity is highly prevalent among nursing home residents with dementia (den Ouden et al., 2015; Moyle et al., 2017) and is associated with reduced quality of life and decreased engagement in daily activities. Studies using sensing technologies to classify physical activity in this population have found that most behaviors are sedentary, such as sitting, lying in bed, or related to eating (Huang et al., 2023). Higher levels of daytime physical activity have been linked to better sleep efficiency and fewer nighttime awakenings (Huang et al., 2023). Accurate monitoring of daytime and nighttime activity patterns is therefore essential for designing effective interventions to support healthier sleep–wake cycles and overall wellbeing in this population.

### 1.1 Study aim and objective

This study aims to advance the understanding of the utility of multi-modal sensing technologies for capturing clinically relevant information about sleep and physical activity in people with dementia residing in a nursing home. We would like to note that the study’s objectives did not include validation of the included sensing technologies or comparison to proxy-rated assessment tools. This study’s primary objective was to explore if sensing technologies, such as smartwatches and radar, can characterize subtle variations in sleep quality and physical activity levels between nursing home residents prescribed nightly psychotropic drugs for sleep disturbance with short half-lives, those prescribed psychotropic drugs with long half-lives, and those not receiving nightly psychotropic drugs. Further, the study sought to align with the real-world conditions under which such technologies would be applied within a nursing home setting.

## 2 Methods

### 2.1 Design and study population

**FIGURE 1:**
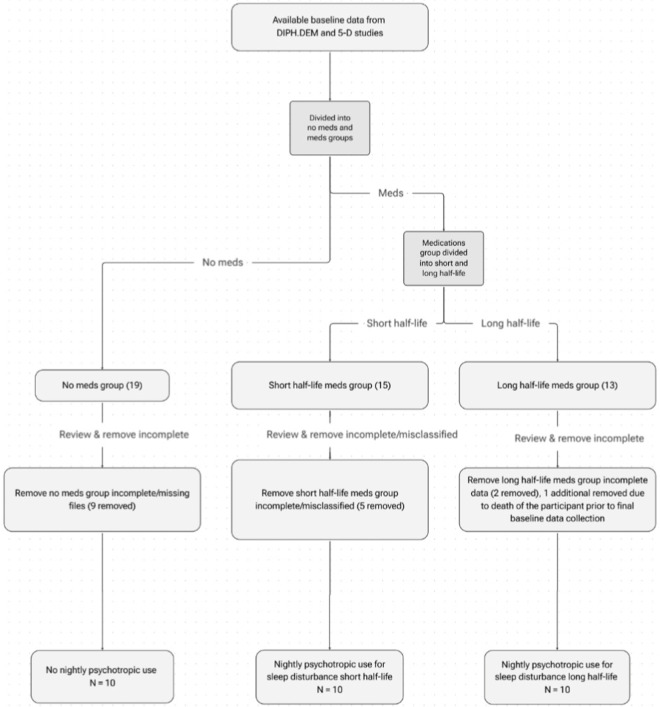
Flow diagram of sampling techniques.

This study used data from two related prospective exploratory studies using observational methods: Digital Phenotyping for changes in activity at the end of life in people with Dementia: an observational trial based on sensing technology (DIPH.DEM) (Boyle et al., 2025) (REK: 634938/ClinicalTrials.gov: NCT06032091) and Decoding Death and Dying in people with Dementia by Digital thanotyping (5-D) (REK: 657596 (NEM 2023/166/CLinicalTrials.gov: NCT06437132). This cross-sectional study is directly related to the DIPH.DEM study, which is a pilot study informing the 5-D study regarding feasibility, methodology, and the potential for clinical use of sensing technologies for people with dementia residing in a nursing home. This study used purposive sampling from DIPH.DEM and 5-D baseline data, collected from February 2024- January 2025, and follows recommendations within previous literature for the use of purposive sampling within quantitative studies (Memon et al., 2025). All participants (N = 47) which had completed baseline data collection from the DIPH.DEM and 5-D cohorts were eligible for inclusion. Initial stratification involved separating the participants into two distinct groups: 1) on nightly medications for sleep disturbance and 2) not on nightly medications for sleep disturbance. From there the group of participants taking nightly psychotropics for sleep disturbance were divided into two further distinctions: 1) those taking drugs with short half-lives, and 2) those taking drugs with long half-lives. Further information about the sampling method is available in the results section and in Figure 1.

### 2.2 Sample size and recruitment

This study is explorative and descriptive, resulting in pragmatic estimation of sample size. Based upon the estimated amount of data acquisition points produced by the included sensing technologies, guidelines for sample sizes within pilot studies (Kunselman, 2024), and recommendations for use of digital biomarkers within analyses (Al-Mekhlafi et al., 2020), we determined that a minimum of thirty participants should be included, with at least ten in each stratified group, and with a minimum of fifty observations for analysis per explored digital biomarkers. Considering these guidelines, we determined that this would be sufficient to give insight into our proposed objectives. Further description of the pilot study, DIPH.DEM, and the primary study, 5-D, methodologies can be found within a previously published proof-of-concept article exploring feasibility of the use of sensing technologies for people with dementia living in a nursing home and the relationship between daytime activity levels and sleep quality (Boyle et al., 2025).

Recruitment was conducted within four nursing homes in Bergen, Norway. Participants were eligible for inclusion to the DIPH.DEM and 5-D studies if they had 1) lived in the nursing home for >6 weeks, were 2) greater than 64 years old, and 3) had a likely diagnosis of dementia or moderate to severe cognitive impairment based upon medical records and results from the baseline administered Clinical Dementia Rating Assessment (CDR) (≥0.5) (Morris, 1997). Exclusion criteria was 1) current presence of delirium at baseline assessment (4A’s Test for Delirium >3) (Tieges et al., 2021), 2) if the participant was currently in an active dying phase, or otherwise considered by their healthcare team to have <8 weeks to live, and 3) if the person had no cognitive impairment or dementia as indicated by the CDR (<0.5) at baseline.

### 2.3 Psychotropic drugs and stratification of participants

Anatomical Therapeutic Chemical (ATC) classifications (Gunnarsdot et al., 2021) for each participant were reviewed prior to the stratification of groups based on nightly psychotropic drug use. Participants taking at least one nightly medication indicated for sleep disturbance noted in their ATC record were classified as taking nightly psychotropic medication. Groups were further classified for participant selection into 1) no nightly medication indicated for sleep disturbance, 2) nightly prescription of psychotropic drugs with a short half-life (i.e.: Melatonin; half-life ≤5 h), and 3) nightly prescription of psychotropic drugs with a long half-life (i.e.: antidepressants, half-life >5 h). The half-life of a drug is defined as the time it takes for a drug within the body to reduce by half. For study purposes we separated psychotropic medications into short (≤5 h) and long (>5 h) half-life categories. Daytime and on-demand medications were not included in the participant selection or group stratification processes.

### 2.4 Outcome measures: questionnaires

Traditional outcome measures used in this study were the Neuropsychiatric Inventory–Nursing home version (NPI- NH) (Selbaek et al., 2008) to assess sleep quality (Nighttime Behaviors section K) and the Physical Self Maintenance Scale (PSMS) to assess activity levels and functional activities of daily living. Traditional questionnaires were answered by proxy within the same week that digital measures were collected by an onsite healthcare representative. The Norwegian version of NPI-NH was found to be reliable and valid (Saari et al., 2022; Wood et al., 2000). Section K of the NPI-NH includes questions about difficulty sleeping, waking up frequently in the night, wandering, taking clothes on in the middle of the night, and waking too early to begin the day. PSMS was developed by Lawton and Brody (1969), Self-maintenance (1969), Edwards (1990) and includes sections for functional activities of ambulation, bathing, grooming, dressing, toileting, and feeding. To precisely define the severity and level of dysfunction within this study, we chose to use a global scoring system from 6 to 30 (6 questions and 5 answers); with lower scores indicating lower dysfunction, and higher scores indicating higher dysfunction. We define the following score ranges regarding independence/activity levels as: 6 = independent, 7–12 = supervision-minimum assist, 13–18 = minimum assist, 19–24 = moderate assistance, 25–30 = max assistance.

Other baseline measurements included the General Medical Health Rating (GMHR) (comorbidities) (Lyketsos et al., 1999), 4AT Test (Morris, 1997), and Clinical Dementia Rating (CDR) (Tieges et al., 2021). GMHR (range 1–4) assesses the number and impact of comorbidities (1 = very good; 2 = good; 3 = moderate, and 4 = bad health) (Lyketsos et al., 1999). The 4AT Test (range 0–12) is a screening tool for delirium and consists of four questions evaluating alertness, cognitive function, and acute change, with a score of four or above indicating potential delirium (Morris, 1997). The CDR (range 0–3) is a widely accepted global scale developed to clinically identify the presence of dementia and stage its severity (score of ≥0.5 - cognitive impairment/likely dementia; 1-mild, 2-moderate, 3-severe dementia) (Tieges et al., 2021). Within our study, participants were included with a score greater than or equal to 0.5 on the CDR. All instructions and traditional questionnaires were provided in the Norwegian language. The nursing home staff administering the data collection tools was trained prior to data collection by the research team.

### 2.5 Sensing technologies and digital biomarkers

The sensing technologies used were Somnofy (Vital Things AS, Trondheim, Norway) (Toften et al., 2020) version 0.7 with sleep algorithm 1.0, Garmin Vivoactive5 (Garmin Ltd., Switzerland) smartwatch firmware version 11.14 (DIPH.DEM) (Kastelic et al., 2021), and Garmin Venu3 firmware version 12.11 (5-D) (Evenson and Spade, 2020). Digital biomarkers reported in this study were acceleration-based physical activity estimates using Euclidean Norm Minus One (ENMO), sleep efficiency (SE), Wake After Sleep Onset (WASO), Sleep Regulatory Index (SRI), Total Sleep Time (TST), no presence (time out of bed), and Sleep Fragmentation Index (SFI) (Table 2).

Somnofy uses contactless radar technology to collect information about sleep cycles, quality of sleep, respiration, air and light quality, and movement during sleep. All lights and audio alerts were turned off, and the devices were connected to a power source within the participant’s room. The average sampling rate for Somnofy is 23.8 Hz with 30 s epochs mimicking gold standard PSG. Somnofy has been validated against PSG in an adult Norwegian population (19–61 years, mean age 28.9 years, SD 9.7) (Toften et al., 2020) with epoch-by-epoch analyses reflecting sensitivity and specificity of 0.97 and 0.72 for Somnofy compared to 0.99 and 0.85 for PSG.

Garmin Vivoactive5 (4 GB) and Garmin Venu3 (8 GB), wrist-worn commercial smartwatches were used to collect information about acceleration (activity levels day/night/24 h). Both devices included an accelerometry sensor which the studies used for collection of raw acceleration data. The key differences regarding inter-comparability between the two smartwatch models are the inclusion of additional sensors in Venu3, barometric altimeter and gyroscope, and the newer heart rate sensor Elevate V5, compared to the previous generation V4 sensor. For the purposes of this study, only the raw data from the accelerometer was used for analysis. Data frequency and collection procedures were the same for both DIPH.DEM and 5-D. The 5-D study used Garmin Venu3 as an upgraded recommendation based upon experiences during the DIPH.DEM study to improve battery life and storage capabilities during data collection periods for the larger 5-D study. Parameters for the smartwatches were synced, paired, and retrieved using a hub through a cloud-based data platform, Fitrockr (Khaled et al., 2022) (Berlin, Germany), with the following settings: acceleration frequency sampling (Fs): 25 Hz. Participants were asked to wear the smartwatch on their preferred wrist 24–7 for 7 days, including bathing and sleeping.

Garmin wearable devices, including the Vivoactive and Venu models, have demonstrated acceptable validity and strong inter-device reliability for step count and movement assessment across laboratory and free-living conditions (Evenson and Spade, 2020; Fuller et al., 2020; Oleh and Obermaisser, 2025). Prior studies support accurate step measurement across walking speeds and age groups, including older adults and slow-paced walking, with excellent agreement observed in adults aged 70–90 years (Höchsmann et al., 2018; Martinato et al., 2021). Garmin Vivoactive devices have also been shown to reliably estimate physical activity and movement using raw acceleration data (White et al., 2024). Further, wrist-worn placement with Garmin deviced using ENMO-derived thresholds for classification of sedentary activities produced superior sensitivity and specificity of sedentary time and light physical activity compared to the classification of moderate-to-vigorous physical activity (Migueles et al., 2021).

### 2.6 Ethics and presumed consent

Potential participants, their next-of-kin, and their primary health contacts at the nursing home were provided with informational materials describing the methods, procedures, and confidentiality of the study. Both DIPH.DEM and 5-D were approved by the Regional Committee for Medical and Healthcare Research Ethics (REK) and the 5-D study additionally by the National Research Ethic Committees (NEM) in Norway: 634938/657596 (NEM 2023/166). Full details of the studies can be found on ClinicalTrials.gov (identifiers: NCT06032091/NCT06437132). Presumed consent from a next-of-kin was obtained if the participants were unable to give informed consent because of dementia.

## 3 Data extraction and preprocessing

The data analysis, extraction and preprocessing, both for acceleration and digital sleep biomarkers, used in this study, were adapted from those used in the previously published DIPH.DEM proof-of-concept study (Boyle et al., 2025). Smartwatches were put on the participant by the researcher on day one of data collection, removed one to two hours for charging on day four by the onsite healthcare contact at the nursing home, and finally removed for data collection day seven by the researcher. Radar devices were installed by the researchers, placed on the wall above the bed in each participants’ private room and connected to a power source. Data from the sensing technologies were collected continuously for 7 days/6 nights.

### 3.1 Acceleration

The standard Garmin consumer application does not provide direct access to raw acceleration data. For research purposes, however, these raw data streams can be extracted through a dedicated research data broker. In this study, raw acceleration and related parameters were obtained using the Fitrockr platform (Berlin, Germany) (Khaled et al., 2022). For each participant, raw acceleration (25 Hz) was exported from the Fitrockr hub/platform in the Comma-Separated Values (CSV) format. Data was downloaded and converted into MATLAB compatible formats for further data processing and cleaning. Using the Unix timestamps, the series were divided into two segments: a 14 h daytime window (07:00:00–20:59:59) and a 10-h nighttime window (21:00:00–06:59:59). The initial and final incomplete days were removed, leaving only full day recordings for analysis. Participants were excluded should they have less than 24-h acceleration data available for analysis. Euclidean norm minus one (ENMO) was calculated using standard formulas in MATLAB. The ENMO is the norm of the three raw acceleration signals from which we have subtracting the gravitational component (1 g = 9.81 m.s^−2^).

$$ENMO_{i}=\sqrt{{{x\left(t\right)}_{i}}^{2}+{{y\left(t\right)}_{i}}^{2}+{{z\left(t\right)}_{i}}^{2}\;}-1000$$

With ${x\left(t\right)}_{i}$ , ${y\left(t\right)}_{i}$ and ${z\left(t\right)}_{i}$ the accelerations on the three axes of the $i^{th}$ datapoint of the sample considered in milli-g.

We considered respectively as daytime, nighttime and daily 24-h the 07:00:00–20:59:00, 21:00:00–06:59:59, and 00:00:00–23:59:59 periods.

### 3.2 Sleep digital biomarkers

Sleep parameters (SE, SRI, WASO, time in no presence, SFI) were exported from the Somnofy platform. The sleep related data from Somnofy was manually inspected to remove daytime sleeping (naps) over the hours of 07:00:00–20:59:00. Naps recorded within the Somnofy data were removed and tallied separately as to not affect the analysis for nighttime sleep totals. Daytime sleeping is not reported within this article as the radar technology only captures sleeping patterns within the sensor range (in bed), neglecting possible daytime sleeping in other areas of the nursing home. The nighttime window was selected to reflect the typical nightly administration of psychotropic medications prescribed for sleep disturbance (noted in the participant’s journal as approximately 21:00) and the routine bedtime procedures in the nursing homes. Other evening medications not indicated for sleep disturbance were administered between the hours of 18:00–21:00. Participants were excluded if they had less than 10 h (1 designated nighttime period) of total sleep data available for final analysis.

SE is the ratio of total sleep time (TST) and total time in bed. The calculation for TST in SE includes sleep onset latency + TST + time awake after sleep onset before final awakening + time attempting to sleep after the final awakening (Kastelic et al., 2021). SE was reported within the Somnofy base data and is scored as a percentage. SRI was also calculated and provided by Somnofy and quantifies the day-to-day similarity of sleep-wake patterns, requiring at least four consecutive nights of data, yielding values ranging from 0 (completely random patterns) to 100 (perfect regularity). WASO is the total number of minutes that a person is awake after having initially fallen asleep (post sleep latency). The SFI is the ratio of the number of shifts from deep sleep to light sleep by total sleep time in hours, higher percentages indicating greater fragmentation of sleep. Somnofy base data additionally provided a metric titled “no presence” which is the amount of time which the participant was outside of the sensor range (defined in the study as >3 m). For purposes of this study, the “no presence” digital biomarker is considered as the time out of bed during nighttime sleep hours. We defined this marker based upon the standard room set up at the nursing home, the standard size of each room, and amount of distance required for the person to be considered out of the bed (>3 m). Sleep awake periods from Somnofy data were manually reviewed over concomitant periods of “no presence” to confirm the accuracy of the data.

### 3.3 Statistical analysis

Descriptive statistics and narrative synthesis are provided for the groups including mean, median, min, max, and standard deviation for all relative variables. Crude estimates for the cohort (N = 30) vs. the three stratified groups (N = 10) are provided to the reader as a comparison and indication of adjustment for gender, age, comorbidities, time living in the nursing home, and disease stage. Data was tested for normality and analyzed using MATLAB R2023a and STATA SE18.5; outliers were detected using regression and box plots. Because the data was non-parametric and not normally distributed, the Kruskal-Wallis rank test was used to test for differences between the three groups. Dunn’s Pairwise Comparison with Bonferroni adjustment was further used as a post hoc evaluation of the specific differences in which the null hypothesis was rejected. Results were considered statistically significant at a p-value <0.05, and at a chi^2^ (2 degrees of freedom) critical value of ≥5.991.

## 4 Results

Baseline data from the DIPH.DEM and the ongoing 5-D study was used for sampling and participant selection purposes within this cross-sectional study. An initial sample of forty-seven participants with a mean age of 86 years old (70–100 years), and 66% female (31 female/16 male), were initially selected (DIPH.DEM: N = 11; 5-D: N = 36). Participants were stratified by medication status into no medication (N = 19) and taking nightly psychotropic medication for sleep disturbance (N = 28) groups. The medication group was further stratified by pharmacokinetic half-life into short half-life (N = 15) and long half-life (N = 13) categories.

One participant was excluded due to death and absence of a corresponding digital data file, resulting in 46 participants eligible for review. Data quality checks and application of inclusion and exclusion criteria led to additional exclusions due to missing or incomplete proxy-rated records, inability to process JSON sleep data files, incomplete medication reviews, duplicate records, or medication misclassification. Following these exclusions, the final analytic sample comprised thirty participants, distributed across three groups: no medication (N = 10) (Windred et al., 2024), short half-life medication (N = 10), and long half-life medication (N = 10); a flow chart representing the sampling process is presented for the reader in Figure 1.

Participants were excluded if they did not meet inclusion criteria and/or had incomplete data profiles (i.e.: less than 24-h acceleration, less than one night or 10 h sleep data, or missing scores from questionnaires or complete medication profiles). The reasons for missing or incomplete data and subsequent drop outs from the study included the following: data quality, device malfunction, clarity of translated data, adherence (removal of smartwatch), death prior to total data collection, behavioral disturbances during data collection (i.e.: participant out of sensor range due to nighttime BPSD), no or only partial medication history provided, and/or missing data from traditional questionnaires at baseline. After the final exclusion of seventeen participants based upon missing or incomplete data profiles, thirty total participants were included (N = 10 from DIPH.DEM, N = 20 from 5-D), and ten total participants were included in each of the three strata for final analysis (Table 1). Furthermore, a total of 1,119 total sleep observations, including 241 total nights, were used in the final analysis of the sleep data; and approximately 1.27*10^8^ observations for the analysis of the acceleration data.

**TABLE 1:** Baseline characteristics within the cohort (N = 30) and within stratified groups (N = 10): short half-lives, long half-lives, and no nightly use of psychotropic medication (None).

Characteristic	Crude estimates (N = 30) mean, median, SD, range	Short half-life (N = 10)	Long half-life (N = 10)	None (N = 10)
Female, n (%)	21 (70)	7 (70)	7 (70)	7 (70)
Age (years)	85.6, 86, SD ± 5.7, 73–100	87.6, 87.5, ±4.3, 80–94	86.7, 86, ±5.7, 79–100	82.4, 82.5, ±6.0, 73–93
Living in nursing home (months)	18.7, 15, ±14.2, 3–84	14.0, 13.5, ±2.5, 11–18	24.0, 17.5, ±23.0, 3–84	18.3, 18, ±7.6, 8–36
Comorbidities (GMHR)	2.8, 3, ±0.7, 1–4	2.5, 3, ±0.7, 1–3	3.0, 3, ±0.5, 2–4	2.9, 3, ±0.7, 1–4
CDR	2.0, 2, ±0.6, 1–3	1.73, 1.5, ±0.5, 1–3	2.0, 2, ±0.7, 1–3	2.2, 2.1, ±0.5, 1–3
NPI-NH				
Delusion	3.8, 4, ±2.5, 1–8	2.0, 2, ±1.4, 1–3	6.7, 6, ±1.2, 6–8	2.5, 2.5, ±1.7, 1–4
Hallucination	2.7, 2, ±1.8, 1–6	1.5, 1.5, ±0.7, 1–2	3.0, 3, ±1.0, 2–4	3.5, 3.5, ±3.5, 1–6
Agitation	2.4, 2, ±2.1, 1–6	4.0, 4, ±2.8, 2–6	2.5, 1.5, ±2.4, 1–6	1.3, 1, ±0.6, 1–2
Depression	2.8, 2, ±3.1, 1–12	3.0, 4, ±1.7, 1–4	3.5, 2, ±4.2, 1–12	1.5, 1.5, ±0.7, 1–2
Anxiety	2.6, 1.5, ±2.5, 1–9	3.3, 3, ±2.5, 1–6	2.8, 1.5, ±3.1, 1–9	2.0, 2, ±0, 2–2
Euphoria	5.7, 6, ±2.5, 3–8	6.0, 6, ±0, 6–6	5.5, 5.5, ±3.5, 3–8	N/A
Apathy	4.6, 3, ±3.5, 1–8	4.0, 4, ±2.8, 2–6	4.3, 3, ±3.2, 2–8	2.5, 2.5, ±2.1, 1–4
Disinhibition	1.8, 1.5, ±1.2, 1–4	N/A	1.3, 1, ±0.6, 1–2	3.0, 3, ±1.4, 2–4
Irritability	3.5, 3, ±2.5, 1–8	4.0, 4, ±2.8, 2–6	4.7, 4, ±3.1, 2–8	2.5, 2.5, ±2.1, 1–4
Aberrant motor	3.0, 2.5, ±, 1–6	6.0, 6, ±0, 6–6	2.0, 1, ±1.7, 1–4	2.5, 2.5, ±2.1, 1–4
Sleep	4.8, 5, ±2.5, 1–9	3.3, 3, ±2.5, 1–6	5.8, 5, ±2.2, 4–9	4.3, 6, ±2.9, 1–6
Appetite	6.6, 6, ±3.6, 3–12	N/A	9.0, 9, ±4.2, 6–12	5.0, 4, ±2.6, 3–8
PSMS	18.6, 18, ±5.2, 8–28	16.1, 16, ±3.5, 10–23	21.4, 21.5, ±5.9, 10–28	18.3, 18, ±5.0, 8–26
Total medication	7.5, 7, ±2.8, 2–13	7.6, 7.5, ±2.3, 4–11	9.0, 8.5, ±2.8, 5–13	6.4, 6, ±3.1, 2–13
Psychotropic use	1.7, 1, ±1, 1–5	1.6, 1.5, ±0.7, 1–3	1.7, 1.5, ±0.9, 1–4	1.3, 1, ±0.5, 1–2

Acronyms and score explanations: The General Medical Health Rating (GMHR) has a 1-4 score indicating somatic health that is (1) very good, (2) good, (3) moderate, and (4) bad. These scores are classified based upon the number of diagnoses/somatic issues and daily medication use. Clinical Dementia Rating (CDR) scores range from 0–3 with a score of >0.5 (1-mild, 2-moderate, 3-severe) indicating likely dementia. Score averages were made based on a total global sum/6 (total questions). Final scores, classifications and min/max totals are based on an algorithm (Supplementary Material), which weights the first section for memory higher than the preceding sections. The Neuropsychiatric Index–Nursing Home version (NPI-NH) features twelve symptoms/behaviors and has a score ranging 1–12 within each section, with higher scores indicating more disfunction. The Physical Self Maintenance Scale (PSMS) measures five constructs of functional daily living and is scored ranging from 6–30 with higher scores indicating higher levels of disfunction. Abbreviations: N/A; not applicable indicating a score of zero; SD, standard deviation.

Participants included in the final analysis (N = 30) had a mean age of 86 years (73–100 years) (Table 1). The CDR assessment showed a mean score of 2, indicating moderate dementia within the cohort. Comorbidities ranged from 1–4 with a mean in each group of approximately 3, indicating moderate health status and greater than three unstable conditions, or several stable chronic conditions that require several daily medications to maintain symptoms. According to the proxy rated NPI-NH (K), 37% of the participants had present nighttime behaviors at baseline. Similar PSMS scores were observed within the cohort (range from 8–28) indicating a need for minimum to moderate assistance with functional activities of daily living.

Drug half-lives ranged from 20 min to 80 h (S1). The following medication categories were identified within the cohort as psychotropic drugs prescribed and taken nightly for sleep disturbance (short and long half-lives): sedatives and antidepressants (ATC codes N05C and N06A), with the short half-life group consisted of sedatives N05C H01 and N05C F01, and the long half-life group consisted of antidepressants with ATC code N06A X11. In addition, the following medication categories were observed as secondary medications across all groups with potential for compounding sedative side-effects (drowsiness, sleepiness, impaired cognition, etc.) being taken nightly between the hours of 18:00–21:00, however, were not specifically prescribed for sleep disturbances: anxiolytic, antipsychotics, analgesics, antidementia, dopaminergic, and antiepileptics (S1). The mean for nightly medications for those prescribed medication for sleep disturbance, including both psychotropic drugs and secondary medications with sedative side-effects, was approximately two per participant (1.7), ranging from 1-4 medications total (Table 1). Although use of psychotropic medications was similar between the stratified groups (mean range 1.25–1.7), overall total medication use was 40% greater in the group taking psychotropic medication with a long half-life vs. the group not taking nightly psychotropic drugs (mean ratio of 9:6.4) (Table 1).

**FIGURE 2:**
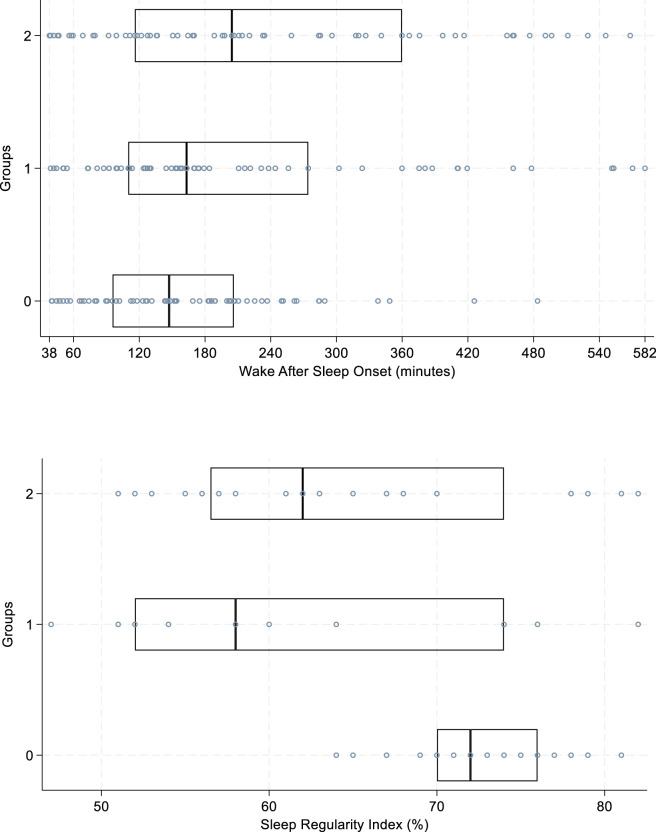
Box plot with included scatter plot of all data points. Sleep regulation (%) and awake time (minutes); Kruskal-Wallis rank test and Dunn’s Pairwise Comparison with Bonferroni adjustment (post hoc evaluation), differences between groups. Definitions: y-axis lists groups 0–2: Group 0 = no nightly psychotropic use, Group 1 = nightly psychotropic use with a short half-life, Group 2 = nightly psychotropic use with a long half-life; horizontal line within the box represents the median; medium-blue dots represent all data points observed in analyses. Sleep Regularity Index is presented as a % from 0%–100%, with 100% being a perfect score. Wake After Sleep Onset is presented from the minimum to maximum (38–582) wake time in minutes; hourly points are noted between the minimum and maximum totals as a reference.

**FIGURE 3:**
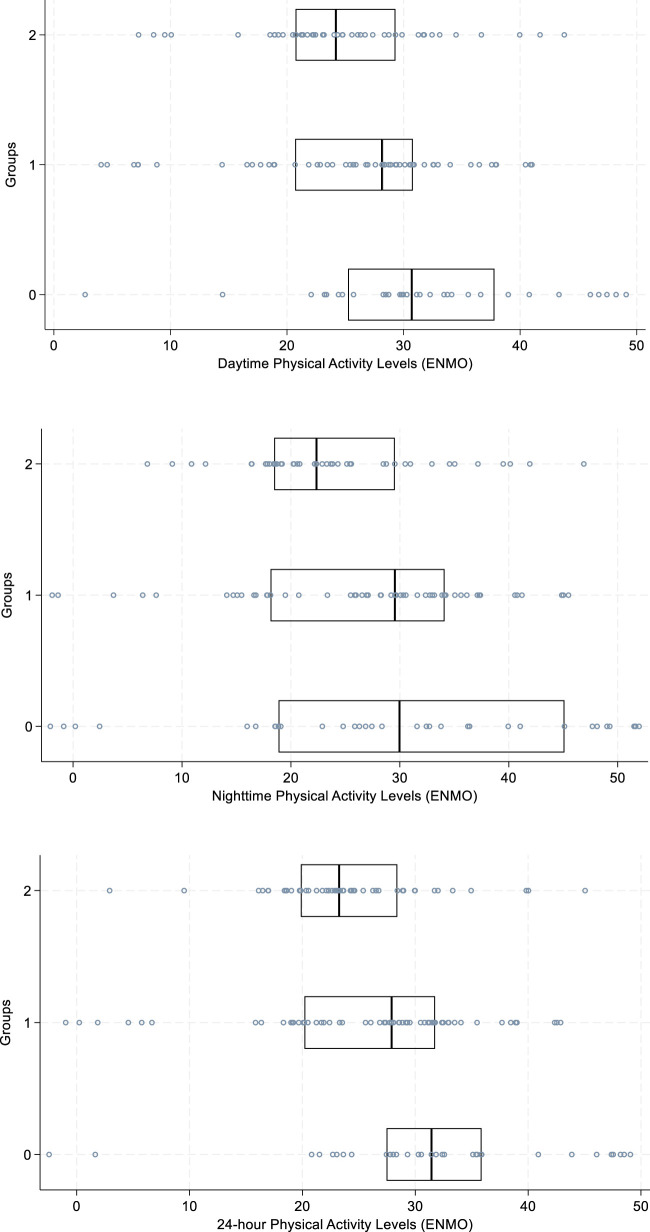
ENMO (milli-g) day/night/24-h; Kruskal-Wallis rank test and Dunn’s Pairwise Comparison with Bonferroni adjustment (post hoc evaluation), differences between groups. Abbreviations: ENMO; Euclidean Norm Minus One. Definitions: y-axis lists groups 0–2: Group 0 = no nightly psychotropic use, Group 1 = nightly psychotropic use with a short half-life, Group 2 = nightly psychotropic use with a long half-life; horizontal line within the box represents the median; medium-blue dots represent all data points observed in analyses. ENMO is presented in milli-gravitational units (milli-g).

Differences in WASO (p = 0.01) (Figure 2) were found and those on nightly psychotropic medications for sleep disturbance had more nightly awakenings. The greatest differences were observed in the participants in the long half-life group (group 2), with a WASO score that was 62% greater than those not taking nightly psychotropic drugs (p = 0.006). Differences were also observed in SRI between both groups taking nightly psychotropic drugs (groups 1/2) and the group not taking nightly psychotropics (p = 0.0001) (Figure 2), indicating that the groups within the cohort taking nightly psychotropic medications for sleep disturbance had less regularity in their sleep cycles from day-to-day. A sensitivity analysis was performed as a sub-analysis by removing influential extreme outliers which were defined as totals beyond 1.5 times the Interquartile Range (IQR). These outliers can be observed in Figures 2, 3 represented within the overlayed scatter plot (medium-blue dots). After removal of extreme values, both WASO (p = 0.03) and SRI (p = 0.001) sensitivity sub-group analysis results supported the original observations.

Furthermore, differences were found between the groups regarding levels of daytime (p = 0.01), nighttime (p = 0.02), and 24-h (p = 0.002) physical activity levels (Figure 3). The groups on nightly psychotropic drugs (groups 1/2) indicated for sleep disturbance had the lowest combined (24-h) physical activity levels (ENMO milli-g) with the greatest difference (day/night, and 24-h) seen between the group not taking nightly psychotropic medication (group 0) and the group on nightly psychotropic drugs with long half-lives (group 2). Sensitivity analysis was performed as described above for ENMO daytime, nighttime, and 24-h totals with results supporting the original findings for day (p = 0.002), night (p = 0.04), and 24-h (p = 0.001) physical activity levels. Further results from all analyses are reported for the reader in Table 2.

**TABLE 2:** Baseline results from selected traditional outcome measures and digital biomarkers; Kruskal-Wallis rank test and post hoc Dunn’s test.

Measurements	Short half-life (N = 10) mean, median, SD, range	Long half-life (N = 10)	No psychotropics (N = 10)	Kruskal–Wallis χ^2^	p-value	Dunn’s test
NPI-NH (K)	3.3, 3, ±2.5, 1–6	5.8, 5, ±2.2, 4–9	4.3, 6, ±2.9, 1–6	1.53	0.465	–
PSMS (physical activity)	16.1, 16, ±3.5, 10–23	21.4, 21.5, ±5.9, 10–28	18.3, 18, ±5.0, 8–26	2.61	0.271	–
Daytime activity (ENMO, milli-g, 07:00–20:59)	25.8, 28.2, ±9.0, 4.1–41.0	24.7, 24.2, ±8.0, 7.3–43.8	30.1, 30.7, ±17.5, −8.7–63.2	*9.48	*0.009	0.049 (0:1); 0.004 (0:2)
Nighttime activity (ENMO, milli-g, 21:00–06:59)	25.7, 29.2, ±12.8, −9.5–45.5	23.4, 22.3, ±9.7, −3.2–46.9	31.9, 32.4, ±19.4, −4.2–68.7	*12.83	*0.002	0.034 (0:1); 0.0006 (0:2)
24-h Activity (ENMO, milli-g, 07:00–06:59)	25.7, 27.9, ±10.2, −1.0–42.9	24.2, 23.3, ±7.5, 2.9–45.0	31.0, 31.6, ±17.4, −5.4–65.6	*8.23	*0.016	0.0062 (0:2)
Total sleep time (TST, hours)	10.7, 10.3, ±3.9, 3–23	10.6, 10.7, ±5.1, 2–24	11.5, 11.1, ±3.5, 3–23	3.17	0.204	–
Sleep efficiency (SE, %)	74.2, 77, ±14, 44–97	72.8, 74.5, ±16, 30–97	73.6, 75, ±9.9, 50–95	0.46	0.795	–
Wake after sleep onset (WASO, hours)	3.6, 2.7, ±2.4, 0.7–10	4.4, 3.4, ±3.3, 0.5–15	2.7, 2.4, ±1.8, 0.43–10	*8.71	*0.013	0.006 (0:2)
Sleep regularity index (SRI, %)	57.8, 54, ±14, 42–83	65.1, 62, ±10.5, 51–83	73.6, 72, ±6.6, 64–93	*20.63	*0.000	<0.0001 (0:1); 0.004 (0:2)
Sleep fragmentation index (SFI, %)	17.4, 13, ±17, 1–71	23.8, 16, ±24.7, 1–96	17.6, 15, ±16.5, 1–100	0.76	0.682	–
No presence (minutes)	17.9, 16.5, ±13, 0.5–55	17.5, 9.5, ±19.5, 0.5–79	13.3, 10, ±10.3, 0.5–59	3.62	0.164	–

*p-value ≤0.05, or chi^2^ (χ^2^) critical value ≥5.991. Abbreviations: NPI-NURSING HOME (K); Neuropsychiatric Inventory-Nursing home version (section K nighttime behaviors), PSMS; personal Physical Self Maintenance Scale, ENMO; euclidean norm minus one, milli-g; 1/1,000th of a gravitational (g) unit, med; median, SD; standard deviation. Dunn’s test results: 0 = group taking no nightly sedative medication, 1 = group taking nightly sedatives (short half-life), 2 = group taking nightly sedatives (long half-life). Definitions: “No presence” refers to the time out of bed (outside sensor range defined as >3 m) in minutes.

## 5 Discussion

This study sought to explore the utility of using digital biomarkers, collected via multiple sensing technologies, to characterize variations in sleep quality and daily activity levels associated with nightly psychotropic drug use. We found that the selected digital biomarkers from the sensing technologies offered a rich description for sleep quality and physical activity levels enabling detection of significant and distinct differences between the groups. The groups taking nightly psychotropic drugs (short and long half-life) had poorer sleep quality and less physical activity (daytime, nighttime, and 24-h) compared to participants not prescribed medications for sleep disturbances. The participants on psychotropic medications with a longer half-life were awake more often and had poorer sleep regulation. Day and nighttime physical activity were highest in the group not taking nightly psychotropic medications, indicating that these types of interventions may potentially decrease nighttime behaviors (BPSD), however, may also have an impact on daytime sedentary levels. Physical activity levels within all groups were suggestive of a highly sedentary daily environment with pronounced physical inactivity. We caution that interpretation of these results should be viewed conservatively based upon the low sample size, however, further investigation is warranted based on these preliminary results. The results highlight the applicability of sensing technologies for the detection of subtle changes in sleep behaviors and physical activity levels of people with dementia residing in nursing homes.

Age-specific WASO cutoffs for adults >70 years are not firmly established; however, a meta-analysis using gold standard polysomnography and a total of 5,273 participant data concluded that WASO increases with age by approximately 10 min per decade (Iskander et al., 2023). In healthy older adults aged 70 years and above, typical WASO values commonly exceed 60 min per night. Normative values for SRI in adults older than 70 years are also not well established, however, a study using actigraphy from 1,987 older adults (mean age 68.7) reported a mean SRI of ∼71.6 ± 14.5 (%), with higher values (≈84 or above) reflecting more regular sleep–wake patterns (Lunsford-Avery et al., 2018). In another recent study, data from 60,977 UK Biobank participants were used (mean age 62.8 ± 7.8 years), finding a mean SRI percentage of 81.0 (73.8–86.3) and concluding that SRI was a strong predictor of mortality (Iskander et al., 2023). Mean values in this study demonstrated higher WASO and lower SRI than those previously reported, with individuals using psychotropic medications for sleep disturbances showing the longest nightly wake periods and the lowest levels of sleep regulation. Relevant normative values should be further explored and established for people with dementia greater than 70 years of age to improve generalizability of future studies.

Sleep disturbances in people with dementia are common and recommended guidelines (Watt et al., 2024) for treatment state that a first approach should be non-pharmacological, however, pharmacological interventions are often used in management of sleep disorders despite low-certainty evidence of effectiveness and potential adverse side-effects (Lim et al., 2023). In agreement with previous literature (McCleery and Sharpley, 2020; Blackman et al., 2021; Calsolaro et al., 2021), this study found that participants were on multiple (≥2) pharmaceutical interventions. A study by Brimelow et al. investigated psychotropic drug prescription in long-term care facilities finding that as high as one in every two residents were prescribed psychotropic drugs for BPSD, and concluded that over half of the prescribed medications were inappropriate and that residents with dementia were more likely to receive these drugs for agitation and psychosis and less likely for sleep disturbances (De Bondt et al., 2025). Other commonly prescribed psychotropics for treatment of BPSD include antidepressants, anxiolytics, and antipsychotics (De Bondt et al., 2025). ATC codes for sedatives (short half-life) and antidepressants (long half-life) were identified within this cohort as meeting our study definition of prescribed psychotropic drugs for sleep disturbance and subsequently used within the provided analysis. Other psychotropic medications, not indicated for sleep and with varying half-lives, taken between these same nighttime hours were also noted and are provided for the reader (S1) as an indication of the cohort’s complex polypharmacy profile. The potential for compounded effects of polypharmacy should also be accounted for in future studies as this can introduce omission and confounder biases.

Inactivity in nursing homes is also extremely common and increased physical activity levels as a non-pharmacological intervention for people with dementia that have sleep disorders demonstrate positive effects on sleep quality (Wilfling et al., 2023). The dose-response relationship between physical activity levels and sleep quality in healthy older adults has been recommended to be optimal at 440 METs-min/week, equivalent to 160 min of moderate intensity training (Xue et al., 2024), however, the bidirectional relationship and recommendations for older adults with dementia remain unclear and underdeveloped (Sewell et al., 2021). Further, there are no recommendations made in a universal physical activity metric based on raw acceleration data, such as ENMO, for daily minimal physical activity levels for older adults with complex conditions. It should also be noted that physical activity levels measured using acceleration, or other common metrics such as step count, do not necessarily adequately measure physical fitness levels. This emphasizes a potential gap in the current literature and these important differences should be accounted for in future study designs. The results of this study indicate that all groups had high levels of daytime inactivity, meeting or below established thresholds for sedentary activities in older populations (Bakrania et al., 2016). Future research should explore clinically relevant recommendations for exercise, physical activity levels, and physical fitness dosage based on more standardized metrics and on the establishment of the classification of activities relevant to more vulnerable populations, such as people with dementia residing in nursing homes.

Although the traditional assessment instruments used in this study, the NPI-NH and PSMS, are well-established, proxy-rated questionnaires widely employed in real-world nursing home settings to evaluate functional activity and sleep-related behaviors, they do not assess the same sleep quality constructs captured by the included digital measures. Consequently, within this study, specific digital biomarkers of sleep, such as WASO, were aligned with the NPI-NH “nighttime behaviors” domain (Section K) for comparative purposes. Similarly, a global PSMS score was used to facilitate comparison between proxy-rated functional activity levels and accelerometry-derived activity measures. Because these proxy-rated instruments assess different domains and operate over distinct temporal frameworks, they fundamentally capture constructs that are not directly equivalent to those derived from sensor-based measures of sleep and activity. Future studies should therefore consider alternative or complementary assessment tools that are more closely aligned with sensor-derived metrics to enhance construct validity and clinical interpretability.

### 5.1 Limitations

Although this study illustrates the utility of sensing technologies for people with dementia residing in a nursing home, it is not without limitations. First, and importantly, there is the lack of validation of sensing technologies mainly due to the rapid development and obsolescence of devices. This study did not intend to evaluate the performance of the technologies included, however, it should be noted that although previously validated and considered reliable, there is still potential for introduction of systematic biases due to the proprietary differences in sensor characteristics between device models. Furthermore, because the studied cohort had pronounced heterogenous qualities, it would be an advantage in a future study to investigate the risk for decreased sleep quality and physical activity levels in the days after prolonged use of psychotropic drugs within a larger and more homogenous group of nursing home residents with dementia.

This study’s design precludes causal inference, however, we note that daytime medication use, not accounted for in this study, could potentially affect physical activity levels and sleep quality profiles. Another consideration regarding this study’s results is the potential for indication bias; meaning that participants with greater baseline sleep disturbance and/or other severe neuropsychiatric symptoms are preferentially prescribed psychotropic medications with longer half-lives. We would like to note that the medication in this context is not an intrinsic or fixed patient characteristic and may reflect prescriber practices and transient clinical conditions. As this study is cross-sectional, group selection should be understood as a snapshot at the time of measurement rather than a stable exposure, and causal interpretations are therefore not possible. The observed differences between groups, therefore, may reflect a combination of patient characteristics, prescribing decisions, and care practices, which align with the real-world conditions under which such technologies would be clinically applied. This should be accounted for in future studies should investigation of medication effectiveness be considered the primary objective.

Lastly, the sample size was estimated based on methodology for a pilot study (DIPH.DEM), which aimed to explore the feasibility and potential use of sensing technologies for people with dementia residing in a nursing home, and to inform a larger study (5-D) with similar methodology. The stratified groups and amount of digital biomarker observations are of sufficient size for a pilot study design; however, the reader should caution the interpretation of the results based on the sample size. A sensitivity analysis of non-parametric comparisons was performed and reported in the results section of the paper with results which support the reported conclusions, however, we want to note that with smaller sample sizes non-parametric analysis is highly sensitive to individual observations. Regardless, we feel that further investigation is warranted within a larger cohort which will improve the generalization of these results.

## 6 Conclusion

This study found that the selected sensing technologies provided nuanced information about sleep quality and physical activity level changes in people with dementia living in a nursing home environment, resulting in distinction between groups taking both short and long half-life psychotropic medications for sleep disturbances. Sensing technologies may be a promising enrichment to commonly used proxy-rated assessments for measurement of sleep disturbance and physical activity levels. Nonetheless, the clinical use of sensing technologies as assessment tools are currently impeded by challenges such as the rapid development of new devices and subsequent need for new validation techniques, and the need for standardization of metrics to improve generalizability and applicability of results.

## Data Availability

The raw data supporting the conclusions of this article will be made available by the authors, without undue reservation.
