# Supplementary material for: Use of sensing technologies to assess sleep quality and physical activity levels in nursing home residents with dementia taking nightly psychotropic drugs for sleep disturbance: a cross-sectional study
Source: Front Aging. 2026 Feb 25;7:1768935. doi: 10.3389/fragi.2026.1768935 (PMC12975902; doi:10.3389/fragi.2026.1768935)
Supplement: Supplementary file 1 [file Supplementaryfile1.docx]

**SUPPLEMENTAL MATERIAL:
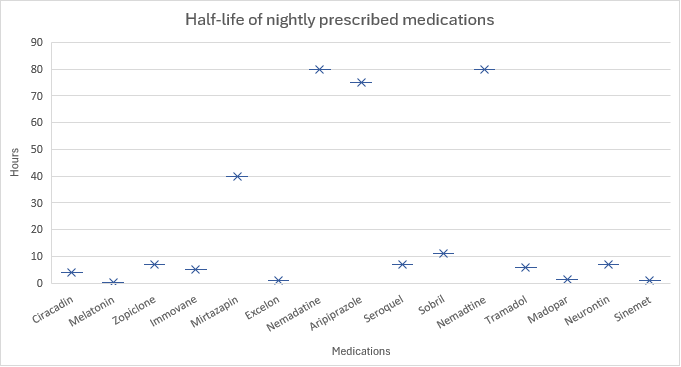
**

**S1.** List of medication half-lives for drugs prescribed nightly (18:00-21:00) within the total cohort (N=30) including psychotropic drugs for sleep disturbance (which were used for analyses) and other related medications with potential sedative side-effects (i.e.: sleepiness, drowsiness, dizziness, confusion); illustrative of the complex polypharmacy and multi-morbidity within the analyzed cohort.
